# Tobacco-driven digestive disease burden: A China–US comparative analysis, 1990–2035

**DOI:** 10.18332/tid/226881

**Published:** 2026-09-21

**Authors:** Zhuo Cheng, Xue Luo, Da Yan, Qiuyu Chen, Han Lang, Yaxin Wu, Hongquan Pang

**Affiliations:** 1 Department of GastroenterologyDazhou Central HospitalDazhouChina; 2 Department of Endocrinology and MetabolismDazhou Central HospitalDazhouChina

**Keywords:** smoking, digestive diseases, Global Burden of Disease, decomposition analysis, Bayesian age-period-cohort model

## Abstract

**Introduction:**

Smoking is a well-established risk factor for digestive diseases, yet direct comparisons of smoking-attributable digestive disease burden between nations with divergent tobacco control trajectories remain scarce. This study compared temporal trends, driving factors, and future projections of this burden in China and the United States from 1990 to 2023.

**Methods:**

Using Global Burden of Disease 2023 data, we analyzed smoking-attributable digestive disease deaths and disability-adjusted life years. Joinpoint regression assessed temporal trends. Decomposition analysis partitioned burden changes into population growth, aging, and epidemiological components. Bayesian age-period-cohort models projected burden to 2035.

**Results:**

From 1990 to 2023, age-standardized mortality rates for smoking-attributable digestive diseases declined by 77.2% in China (average annual percentage change, AAPC= -4.32; 95% CI: -4.42 – -4.21; p<0.001) and 58.0% in the United States (AAPC= -2.57; 95% CI: -2.66 – -2.48; p<0.001). Despite these rate reductions, absolute smoking-attributable deaths among those aged 90–94 years increased by 663.6% in China and 131.5% in the United States. Epidemiological change was the dominant driver of burden reduction, contributing 374.4% and 349.8% of the overall smoking-attributable death reduction in China and the United States, respectively, outweighing combined demographic pressures. Joinpoint analysis identified a transient increase in US smoking-attributable death rates during 2018–2021 (APC=2.51; p<0.05). Projections indicated continued declines in smoking-attributable rates through 2035.

**Conclusions:**

Despite substantial epidemiological improvements, population aging increasingly offsets rate declines, particularly among the oldest adults. Sustained, age-targeted tobacco control strategies are essential to address the evolving demographic burden.

## Introduction

Tobacco use remains the leading preventable cause of death worldwide, responsible for over 175 million deaths and nearly 4.30 billion years of life lost from 1990–2021^1^. While the pulmonary and cardiovascular consequences of smoking are widely recognized, the digestive system represents a critically underappreciated target of tobacco-induced injury. Digestive diseases encompass a broad spectrum of conditions affecting the gastrointestinal tract, liver, and pancreas, collectively imposing a substantial global health burden^2^. Smoking has been causally linked to numerous digestive diseases through multiple pathogenic mechanisms, including alteration of mucosal defenses, hepatic metabolic dysfunction, impaired gut barrier integrity, and modulation of the gut microbiome^3-5^.

Accumulating evidence has established the causal relationship between tobacco exposure and digestive diseases. A comprehensive Mendelian randomization study demonstrated that genetic predisposition to smoking was associated with increased risk of 20 out of 24 gastrointestinal diseases, with 15 associations persisting after adjustment for alcohol consumption^6^. Cohort evidence further revealed that early-life tobacco exposure increased the risk of digestive diseases by 10–32%, with accelerated biological aging mediating 3–20% of these associations^7^. At the population level, a prospective study of 267408 participants demonstrated that adherence to healthy lifestyles including never smoking was associated with a 28% reduction in digestive disease risk^8^. A prior GBD-based analysis reported that global deaths from tobacco-attributable digestive diseases decreased from 52789 to 34061 between 1990 and 2021^9^, and parallel declining trends have been observed for tobacco-attributable esophageal cancer^10,11^, pancreatic cancer^12^, and colorectal cancer^13^.

Despite these advances, critical knowledge gaps persist. Most existing studies relied on the GBD 2021 dataset, lacked direct country-level comparisons between high-burden nations with divergent tobacco control trajectories, and did not integrate joinpoint regression with decomposition analysis and Bayesian projections within a unified analytical framework. Furthermore, the relative contributions of demographic versus epidemiological factors to burden changes remain inadequately characterized at the country-specific level, and sex-stratified temporal trend analyses with projections are lacking^14,15^.

This study aimed to comprehensively characterize and compare the burden of smoking-attributable digestive diseases in China and the United States from 1990 to 2023, with projections to 2035, using data from the GBD 2023. Specifically, we employed joinpoint regression, decomposition analysis, and Bayesian age-period-cohort models to quantify temporal trends, disentangle demographic and epidemiological drivers, and forecast future burden trajectories, stratified by sex and age, thereby providing actionable evidence to inform targeted tobacco control strategies in these two major nations.

## Methods

### Study design and data source

This population-based comparative study analyzed the burden of digestive diseases attributable to smoking in China and the United States of America from 1990 to 2023, with projections extending to 2035. Data were obtained from the Global Burden of Disease (GBD) Study 2023, coordinated by the Institute for Health Metrics and Evaluation (IHME) at the University of Washington^16^. The GBD 2023 provides comprehensive estimates of disease burden across 204 countries and territories, incorporating data from censuses, household surveys, civil registration and vital statistics systems, disease registries, health service utilization records, and other sources through a standardized analytical framework. Mortality estimates were generated using Cause of Death Ensemble modeling, while morbidity estimates employed DisMod-MR 2.1, a Bayesian meta-regression tool. Uncertainty was propagated throughout the estimation process using 1000 draws from the posterior distribution of each quantity, from which 95% uncertainty intervals (UIs) were derived as the 2.5th and 97.5th percentiles. The risk-attributable burden was estimated using a comparative risk assessment framework that combined exposure estimates with relative risks derived from systematic reviews and meta-analyses, applying the counterfactual of a theoretical minimum risk exposure level. As this study exclusively utilized de-identified, publicly available aggregate data, institutional review board approval was not required. This study was conducted in accordance with the principles of the Declaration of Helsinki.

### Disease and risk factor definitions

Digestive diseases were defined according to the GBD cause hierarchy, encompassing conditions mapped to the International Classification of Diseases, Tenth Revision (ICD-10) codes I84–I85.9, I98.2, K15.9–K42.9, K44–K52, K52.2–K62, K62.4–K62.6, K62.8–K63.4, K63.8–K67, K67.8–K68.1, K68.12–K75, K75.2, K75.4–K76.2, K76.4–K90.9, K92–K93, K93.8, K96–K99, N29.0, N49.5, R11–R19.8, R85–R85.9, Z52.6, and Z94.4. The corresponding ICD-9 codes include 455–455.9, 456.0–456.21, 530–530.85, 530.89–536.3, 536.8–538, 540–543.9, 550–551.1, 551.3–552.1, 552.3–553.1, 553.3–558.9, 560–560.39, 560.8–562.13, 564–564.1, 564.5–569, 569.1–569.5, 569.81–572, 572.2–579.2, and 579.4–579.9. The risk factor of interest was tobacco smoking, and the attributable burden was estimated using population attributable fractions derived from the GBD comparative risk assessment framework. Two outcome measures were analyzed: deaths and disability-adjusted life years (DALYs), each expressed as both absolute counts and age-standardized rates per 100,000 population (ASRs). Age-specific analyses were restricted to adults aged ≥30 years, stratified into fourteen five-year age groups (30–34 through 90–94, and ≥95 years), consistent with the age range for which smoking-attributable digestive disease burden is estimated in the GBD framework. All analyses were stratified by sex (both sexes combined, male, and female).

### Statistical analysis

Temporal trends in ASRs from 1990 to 2023 were assessed using two complementary approaches. First, the estimated annual percentage change (EAPC) was calculated by fitting a linear regression model with the natural logarithm of the ASR as the dependent variable and calendar year as the independent variable, with the EAPC derived as 100×(e^β^ - 1), where β represents the regression coefficient for year, and its 95% confidence interval (CI) was obtained from the model. Second, joinpoint regression analysis was performed to identify statistically significant changes in temporal trends, using the Joinpoint Regression Program methodology with a maximum of five joinpoints permitted. The model employed a log-linear specification with the assumption of constant variance. For each identified segment, the annual percentage change (APC) and its 95% CI were estimated, and the average annual percentage change (AAPC) was computed as a weighted average of segment-specific APCs over three periods (1990–1999, 2000–2009, and 2010–2023) as well as the full study period. Statistical significance of APC and AAPC estimates was determined using their corresponding p-values.

Percentage changes in absolute counts and ASRs between 1990 and 2023 were calculated as [(value₂₀₂₃ - value₁₉₉₀)/value₁₉₉₀]×100.

To disentangle the contributions of demographic and epidemiological factors to changes in disease burden, a decomposition analysis was conducted. This method partitions the observed change in the absolute number of deaths or DALYs between 1990 and 2023 into three components: population growth, population aging, and changes in age-specific rates (epidemiological change). The decomposition was applied to those aged 30 years to ≥95 years.

Projections of disease burden from 2024 to 2035 were generated using Bayesian age-period-cohort (BAPC) models. This approach accounts for temporal trends across age groups, calendar periods, and birth cohorts simultaneously within a Bayesian framework incorporating integrated nested Laplace approximations. Population projections used for estimating future absolute counts were derived from GBD demographic forecasts. Projected estimates included both ASRs and absolute numbers, with corresponding 95% prediction intervals.

All statistical tests were two-sided, and a p<0.05 was considered statistically significant. All analyses were performed using R software (version 4.3.2; R Foundation for Statistical Computing, Vienna, Austria). Key packages included *dplyr* (version 1.1.4) for data manipulation, *ggplot2* (version 3.4.4) for visualization, *patchwork* (version 1.2.0) for figure composition, *openxlsx* (version 4.2.5.2) for data export, *scales* (version 1.3.0) for axis formatting, and *MetBrewer* (version 0.2.0) for color palettes.

## Results

### Overall trends and temporal patterns of smoking-attributable digestive disease burden

**Figure 1:**
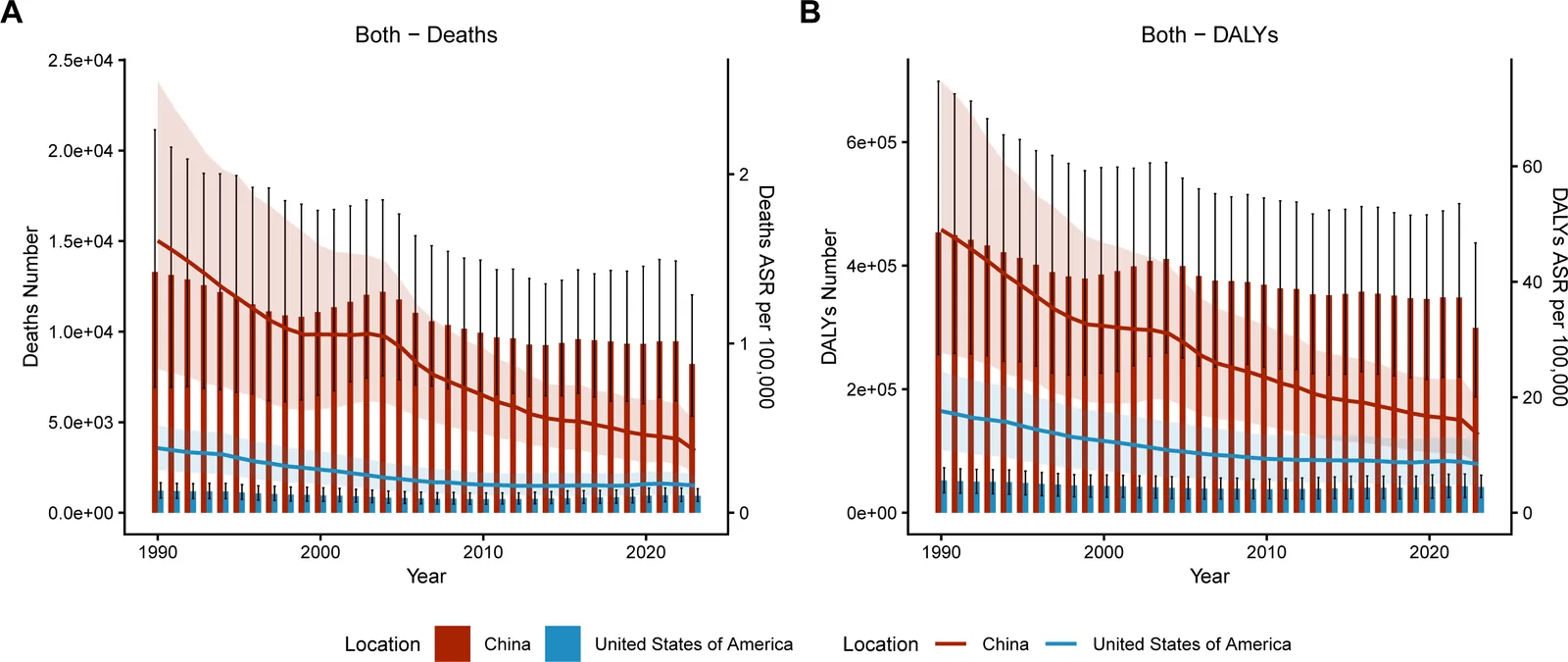
Temporal trends in the number and ASR of smoking-attributable digestive disease deaths and DALYs in China and the United States, 1990–2023: A) Both sexes, deaths; B) Both sexes, DALYs. Bar charts represent the absolute number of cases (left y-axis) with error bars indicating 95% UIs, while line plots with shaded ribbons depict ASR (right y-axis) with 95% UIs

From 1990 to 2023, the burden of smoking-attributable digestive diseases declined in both China and the United States, though substantial between-country disparities persisted in magnitude and pace of change (Table 1, Figure 1A and B; and Supplementary file Figures S1A-S1D). In China, the number of smoking-attributable digestive disease deaths decreased from 13293 (95% UI: 6936–21145) in 1990 to 8217 (95% UI: 5346–12028) in 2023, a reduction of 38.2%, while the ASR for deaths fell from 1.61 (95% UI: 0.85–2.55) to 0.37 (95% UI: 0.24–0.54), representing a 77.2% decline. In the United States, deaths decreased from 1224 (95% UI: 811–1662) to 938 (95% UI: 622–1328), a 23.4% reduction, with the ASR declining by 58.0%. For DALYs, China experienced a 34.1% decrease in absolute numbers and a 72.4% reduction in the ASR, whereas the United States showed a 20.1% decrease in numbers and a 52.1% decline in the rate. Sex-stratified analyses revealed that Chinese females exhibited the steepest decline in the ASR for deaths (85.1%), compared with 76.2% for males and 77.2% for both sexes combined. In the United States, males demonstrated a greater reduction in the ASR for deaths (62.8%) than females (52.4%).

**Table 1:** Overall burden of smoking-attributable digestive diseases in China and the United States, 1990 and 2023

*Country*	*Deaths/* *DALYs*	*Sex*	*Cases in 1990* *n (95% UI)*	*Cases in 2023* *n (95% UI)*	*Change in cases (%)*	*ASR in 1990* *(95% UI)*	*ASR in 2023* *(95% UI)*	*Change in ASR (%)*	*AAPC* *(95% CI)*	*AAPC* *p*
China	Deaths	Both	13293 (6936–21145)	8217 (5346–12028)	-38.19	1.61 (0.85–2.55)	0.37 (0.24–0.54)	-77.2	-4.32 (−4.42 – -4.21)	<0.001
		Male	12079 (6101–19409)	7616 (4931–10976)	-36.95	3.03 (1.52–4.85)	0.72 (0.47–1.05)	-76.16	-4.19 (−4.29 – -4.09)	<0.001
		Female	1215 (514–2570)	601 (337–1096)	-50.51	0.34 (0.15–0.71)	0.05 (0.03–0.09)	-85.11	-5.47 (−5.55 – -5.38)	<0.001
	DALYs	Both	453870 (256284–698541)	299331 (187709–436903)	-34.05	49.03 (27.69–74.73)	13.52 (8.49–19.62)	-72.43	-3.77 (−3.86 – -3.66)	<0.001
		Male	419593 (232781–665291)	278978 (175627–408178)	-33.51	90.59 (50.07–142.76)	25.76 (16.39–37.55)	-71.56	-3.68 (−3.77 – -3.57)	<0.001
		Female	34277 (14425–70149)	20352 (10733–32497)	-40.62	8.25 (3.58–16.91)	1.75 (0.9–2.82)	-78.84	-4.55 (−4.63 – -4.43)	<0.001
USA	Deaths	Both	1224 (811–1662)	938 (622–1328)	-23.37	0.38 (0.25–0.52)	0.16 (0.11–0.23)	-58.02	-2.57 (−2.66 – -2.48)	<0.001
		Male	768 (527–1036)	564 (364–814)	-26.59	0.57 (0.39–0.77)	0.21 (0.14–0.31)	-62.78	-2.93 (−3.01 – -2.84)	<0.001
		Female	456 (284–652)	374 (244–552)	-17.93	0.24 (0.15–0.35)	0.12 (0.07–0.17)	-52.42	-2.2 (−2.3 – -2.09)	<0.001
	DALYs	Both	52321 (32381–72816)	41810 (24559–60836)	-20.09	17.6 (10.85–24.47)	8.44 (4.75–12.47)	-52.07	-2.16 (−2.2 – -2.11)	<0.001
		Male	28422 (18656–38009)	22485 (14045–31829)	-20.89	21.01 (13.79–28.14)	9.23 (5.63–13.28)	-56.06	-2.4 (−2.47 – -2.33)	<0.001
		Female	23898 (13958–34877)	19325 (10408–29739)	-19.14	15.09 (8.75–22.23)	7.78 (4.1–12.09)	-48.43	-1.93 (−1.96 – -1.87)	<0.001

The average annual percentage change (AAPC) with 95% CI and corresponding p-values are derived from joinpoint regression analysis.

ASR: age-standardized rate per 100000 population. CI: confidence interval. DALYs: disability-adjusted life years. UI: uncertainty interval.

ASR: age-standardized rate per 100000 population. UI: uncertainty interval. DALYs: disability-adjusted life years.

Joinpoint regression analysis identified distinct temporal segments in rate trajectories (Supplementary file Figures S2A-S2F). In China, the overall AAPC for the ASR for deaths in both sexes was -4.32 (95% CI: -4.42 – -4.21; p<0.001), with the most pronounced decline occurring during 2021–2023 (APC= -8.87; 95% CI: -10.48 – -6.25; p<0.001) and a period of stagnation during 1999–2004 (APC=0.18; 95% CI: -3.41–1.40; p>0.05). The United States showed a lower overall AAPC of -2.57 (95% CI: -2.66 – -2.48; p<0.001), with a transient reversal during 2018–2021 (APC: 2.51; 95% CI: 1.23–3.33; p<0.05). For DALYs, the AAPC in China was -3.77 (95% CI: -3.86 – -3.66; p<0.001) compared with -2.16 (95% CI: -2.20 – -2.11; p<0.001) in the United States. Chinese females consistently showed the steepest decline, with an overall AAPC of -5.47 (95% CI: -5.55 – -5.38; p<0.001) for ASR for deaths.

### Age-specific burden and temporal trends

**Figure 2:**
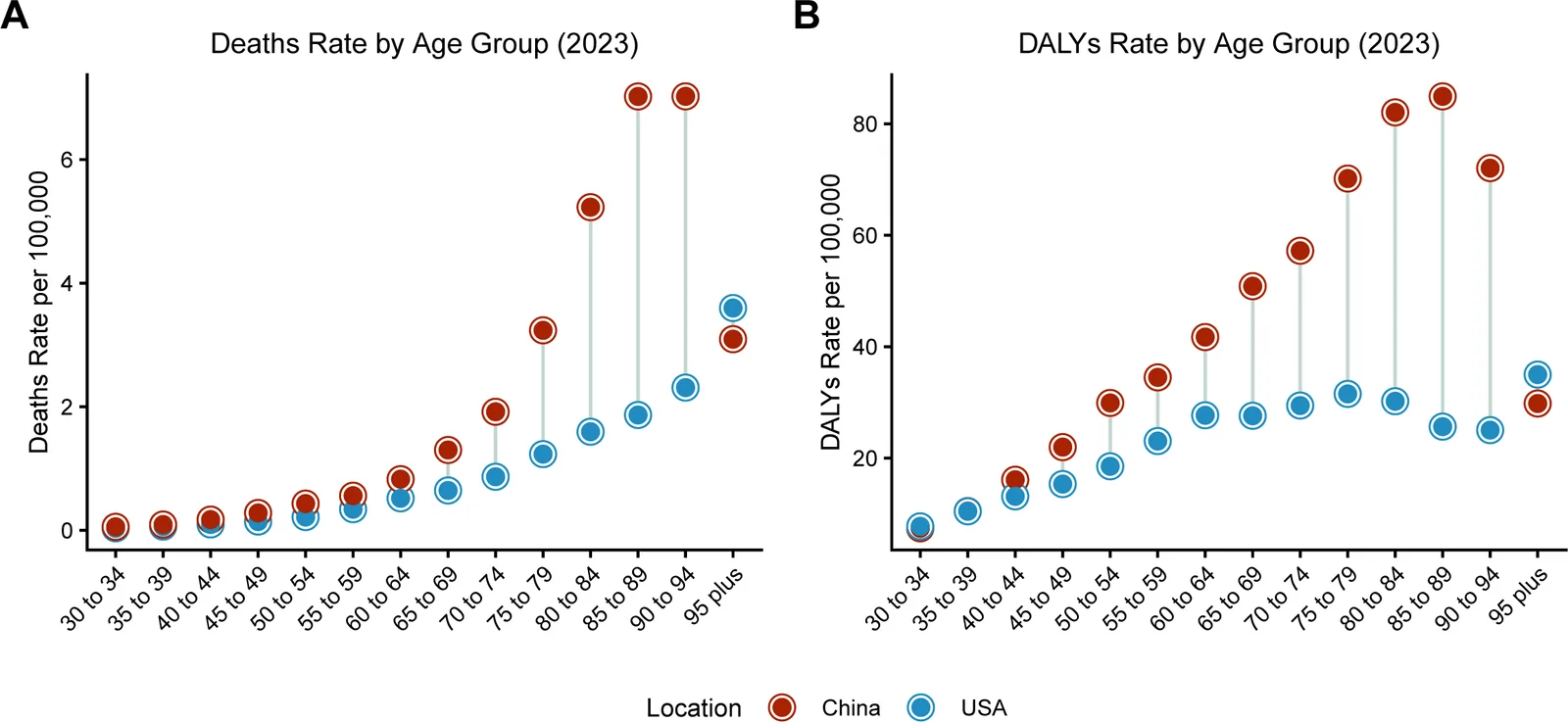
Age-specific comparison of rates of smoking-attributable digestive disease deaths and DALYs between China and the United States in 2023: A) Death rate per 100000 by age group; B) DALYs rate per 100000 by age group. Dumbbell plots display the values for China and the United States across fourteen age groups (30–34 to ≥95 years) in both sexes combined, with connecting lines indicating the magnitude of between-country differences

Beyond the overall declining trends, age-specific analyses revealed heterogeneous patterns across age groups in both countries (Figure 2A and B; and Supplementary file Table S1 and Figures S3A and S3B). In China, the death rate declined across all age groups from 1990 to 2023, with the steepest reductions observed among younger adults: the 30–34 years age group showed an 82.9% decrease in rate (EAPC= -5.48; 95% CI: -5.81 – -5.14), and the 55–59 years age group declined by 84.2% (EAPC= -5.34; 95% CI: -5.54 – -5.14). The ≥95 years age group also exhibited a substantial rate reduction of 82.5% (EAPC= -5.86; 95% CI: -6.51 – -5.21). In contrast, despite declining rates, absolute death counts increased markedly among the oldest age groups: the 85–89 years group rose by 180.8%, the 90–94 years group by 663.6%, and the 80–84 years group by 55.1%. For DALYs, similar divergence was observed, with the 90–94 years age group showing a 703.1% increase in absolute numbers despite a 33.2% reduction in rate (EAPC= -0.47; 95% CI: -0.91 – -0.03).

DALYs: disability-adjusted life years.

In the United States, age-specific death rates generally declined but at a slower pace than in China. The 70–74 years age group experienced a 66.5% rate reduction (EAPC= -3.52; 95% CI: -3.97 – -3.05), while the 30–34 years age group showed a 53.6% decline (EAPC= -1.78; 95% CI: -2.00 – -1.01). Notably, the 90–94 years age group demonstrated virtually no change in death rate (-2.6%; EAPC=0.05; 95% CI: -0.04–0.14), with absolute numbers increasing by 131.5%. For DALYs, the 90–94 years age group was the only group showing an increase in rate (6.8%; EAPC=0.35; 95% CI: 0.25–0.46). In 2023, age-specific rates in both countries consistently increased with advancing age, with the highest death rates observed in the age group of 85–89 years in China (7.02 per 100000) and the ≥95 years age group in the United States (3.60 per 100000).

### Decomposition of changes in smoking-attributable digestive disease burden

**Figure 3:**
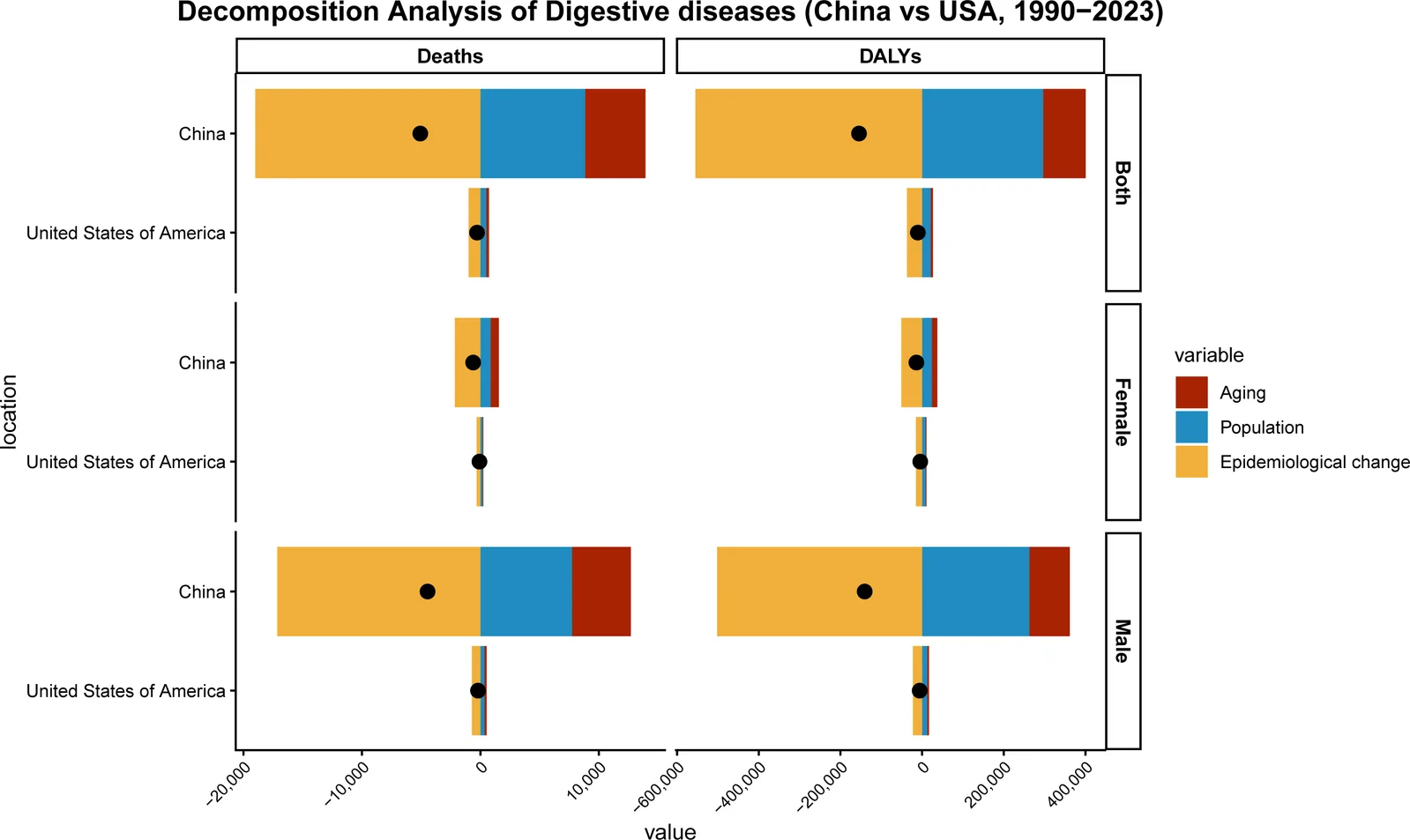
Decomposition analysis of changes in smoking-attributable digestive disease deaths and DALYs between 1990 and 2023 in China and the United States The stacked bar charts decompose the observed change in the absolute number of deaths and DALYs into three components: population growth, population aging, and epidemiological (age-specific rate) change. Results are presented by country, sex (both sexes, male, and female), and outcome measure (deaths and DALYs)

To further elucidate the drivers underlying the observed reductions, decomposition analysis partitioned the changes in absolute burden between 1990 and 2023 into contributions from population growth, population aging, and epidemiological change (Figure 3). In China, the total number of smoking-attributable digestive disease deaths for both sexes decreased by 5076, with epidemiological change contributing a reduction of 19005 (374.4% of the overall difference), which more than offset the increases driven by population growth (+8831; 174.0%) and population aging (+5098; 100.4%). A similar pattern was observed for DALYs: the overall decline of 154539 was attributable to a substantial epidemiological reduction of 554948 (359.1%), counterbalanced by population growth (+296463; 191.8%) and aging (+103946; 67.3%). Sex-stratified results showed that in Chinese males, epidemiological change accounted for a reduction of 17152 deaths (384.3% of the overall difference) and 502022 DALYs (357.0%), while in females, the corresponding reductions were 2169 deaths (353.6%) and 51101 DALYs (367.0%).

DALYs: disability-adjusted life years.

In the United States, the overall number of deaths for both sexes decreased by 286, driven by an epidemiological reduction of 1000 (349.8%), partially offset by population growth (+486; 170.1%) and aging (+228; 79.6%). For DALYs, the total decline of 10511 resulted from an epidemiological reduction of 37022 (352.2%), counteracted by population growth (+20877; 198.6%) and aging (+5635; 53.6%). Among American males, population growth (+12415; 209.1%) exerted a stronger upward pressure on DALYs than aging (+4460; 75.1%), whereas among females, population growth (+8757; 191.5%) similarly exceeded the contribution of aging (+1715; 37.5%). Across both countries and all sex strata, epidemiological change consistently represented the dominant factor driving the net decline in burden, exceeding the combined upward effects of demographic changes.

### Projected burden of smoking-attributable digestive diseases to 2035

Building upon the decomposition findings, Bayesian age-period-cohort models were employed to project the burden of smoking-attributable digestive diseases from 2024 to 2035 (Supplementary file Figures S4A-S4D). By 2035, the projected number of smoking-attributable digestive disease deaths in China for both sexes was estimated at 3646, representing a further substantial reduction from the 2023 level of 8217. The corresponding ASR for deaths was projected to decline to 0.12 compared with 0.37 in 2023. Sex-stratified projections indicated that Chinese males would account for 3133 deaths with a projected ASR of 0.19, while Chinese females were projected to have 513 deaths with a rate of 0.02. In the United States, total deaths for both sexes were projected at 1345, with an ASR of 0.19. American males were projected to have 770 deaths at a rate of 0.22, and females 575 deaths at a rate of 0.14.

## Discussion

### Summary of principal findings

This study comprehensively characterized the burden of smoking-attributable digestive diseases in China and the United States from 1990 to 2023, with projections to 2035. The principal findings demonstrated that both countries experienced substantial declines in ASR for deaths and DALYs over the study period, with China exhibiting steeper reductions (AAPC for death rate= -4.32) than the United States (AAPC= -2.57). Despite declining rates, absolute burden in the oldest age groups increased markedly, particularly in China where deaths among those aged 90–94 years rose by 663.6%. Decomposition analysis revealed that epidemiological change was the dominant driver of net burden reduction in both countries, consistently outweighing the upward pressures from population growth and aging. Joinpoint regression identified distinct temporal inflection points, including a period of stagnation in China (1999–2004) and a transient reversal in the United States (2018–2021). Sex-stratified analyses demonstrated that Chinese females experienced the steepest rate declines, while males bore the overwhelming majority of the absolute burden. Projections to 2035 indicated continued declines in ASRs across both countries.

### Contextualization with existing literature and mechanistic considerations

Our findings are broadly consistent with a prior GBD-based analysis by Qin et al.^9^ which reported that global deaths from tobacco-attributable digestive diseases decreased from 52789 in 1990 to 34061 in 2021, with the ASR declining from 1.34 to 0.40. However, our study extends this work by providing country-specific comparisons between China and the United States using the updated GBD 2023 dataset, incorporating joinpoint regression and BAPC projections that were not performed in the prior analysis. The magnitude of decline we observed in China aligns with a recent comprehensive assessment of tobacco-attributable disease burden across Chinese provinces, which reported an AAPC for age-standardized mortality of -2.34% nationally across all tobacco-related causes^15^. The steeper decline we identified for digestive diseases specifically may reflect the combined effects of tobacco control measures and improvements in gastroenterological care that have been particularly effective for this disease category.

The causal relationship between smoking and digestive diseases is well-supported by multiple lines of evidence. A large-scale Mendelian randomization study by Yuan et al.^6^ demonstrated that genetic predisposition to smoking was associated with increased risk of 20 out of 24 gastrointestinal diseases, with 15 associations persisting after adjustment for alcohol consumption. These findings were further corroborated by cohort evidence from Li et al.^7^ who reported hazard ratios of 1.10 (95% CI: 1.09–1.12) for *in utero* tobacco exposure and 1.32 (95% CI: 1.28–1.35) for childhood smoking initiation in relation to digestive disease risk. The biological mechanisms underlying these associations include alteration of structural, functional, and immunological host defenses^3^, hepatic metabolic dysfunction and impaired gut barrier integrity^5^, and modulation of the gut microbiome favoring carcinogenic bacteria^4^. At the genetic level, tobacco consumption has been shown to significantly increase digestive system cancer susceptibility among carriers of the miR-146a rs2910164 CC/CG genotype (OR=1.39; 95% CI: 1.05–1.84)^17^, while secondhand smoke exposure has been associated with increased ulcerative colitis risk (OR=2.03; 95% CI: 1.03–4.05)^18^.

The divergent temporal patterns between China and the United States warrant careful consideration. The transient increase in ASR for deaths in the US during 2018–2021 coincided with a period when the overall effectiveness of tobacco control may have been partially undermined by the rapid proliferation of e-cigarettes, which accounted for 90.6% of adverse health event reports to the FDA, with the digestive system representing the third most affected category^19^. This temporal pattern is also consistent with evidence that e-cigarettes exert adverse effects on the digestive system through mechanisms distinct from conventional cigarettes^5^. Conversely, the accelerating decline observed in China during 2021–2023 may partially relate to comprehensive tobacco control policies, as modeling studies have demonstrated that accelerated smoking elimination could avert 2.04 billion years of life lost globally by 2050 compared with the reference scenario^1^. The prominent sex disparity we observed, with Chinese males bearing a disproportionately higher burden, is consistent with findings from Yuan et al.^8^ who demonstrated that healthy lifestyle adherence was associated with a hazard ratio of 0.72 (95% CI: 0.70–0.74) for digestive diseases, with never smoking being one of six independently protective behaviors. Our finding that epidemiological change dominated burden reduction aligns with decomposition analyses of smoking-attributable esophageal cancer, which similarly showed population growth (154.62%) and aging (39.75%) driving absolute burden increases while rate improvements offset these demographic pressures^10,11,20^. Analogous patterns have been reported for smoking-attributable digestive cancers in older adults^21^, pancreatic cancer^12^, and colorectal cancer^13^, as well as for tobacco-related neurological disorders and the overall global disease burden attributable to tobacco products^14,22^.

### Strengths and limitations

This study has several methodological strengths. It provides a direct, sex-stratified comparison of smoking-attributable digestive disease burden between China and the United States using the most current GBD 2023 estimates, integrating multiple complementary analytical approaches including joinpoint regression, decomposition analysis, and BAPC projections within a unified framework. However, several limitations should be acknowledged. First, GBD estimates rely on modeling assumptions and data availability that vary by country, potentially introducing differential measurement error. Second, the ecological nature of this study precludes individual-level causal inference. Third, projections assume continuation of historical trends and cannot account for unforeseen policy changes or emerging risk factors. Fourth, the analysis was restricted to adults aged ≥30 years, potentially underestimating the full burden attributable to early-life tobacco exposure, which has been shown to increase digestive disease risk independently^7^. Finally, we could not disaggregate the contribution of specific digestive disease subtypes or distinguish between direct smoking and secondhand smoke exposure, the latter of which contributes meaningfully to the overall tobacco-attributable burden, particularly among females^15^.

### Clinical and public health implications

Our findings carry specific implications for clinical practice and public health policy. The rising absolute burden among the oldest age groups, despite declining rates, underscores the need for enhanced screening and management of smoking-related digestive conditions in geriatric populations, particularly in China, where the ≥80 years age groups showed substantial increases in death counts. The distinct temporal inflection points identified through joinpoint analysis can help evaluate the effectiveness of specific policy interventions and guide future tobacco control efforts. Given that epidemiological change was the dominant factor driving burden reduction, continued investment in tobacco control remains critical, particularly given evidence that the burden attributable to tobacco, alcohol, and obesity in digestive diseases is shifting. Future studies should incorporate individual-level longitudinal data to disentangle the effects of smoking cessation timing, explore the differential impact of conventional versus emerging tobacco products on specific digestive disease subtypes, and evaluate the effectiveness of targeted health warning messages in reducing digestive disease burden. Additionally, the interaction between tobacco exposure and genetic susceptibility warrants further investigation in diverse populations.

## Conclusions

This study demonstrated that the burden of smoking-attributable digestive diseases declined substantially in both China and the United States from 1990 to 2023, driven predominantly by epidemiological improvements that outweighed the opposing effects of population growth and aging. China exhibited steeper rate reductions than the United States, with Chinese females showing the most pronounced declines. However, rising absolute burden among the oldest age groups and distinct temporal inflection points highlight the ongoing demographic challenges and the need for sustained, age-targeted tobacco control strategies. Projections to 2035 suggest continued declines in ASRs, contingent upon the maintenance and strengthening of current tobacco control efforts in both countries.
