# Supplementary material for: Tobacco-driven digestive disease burden: A China–US comparative analysis, 1990–2035
Source: Tob Induc Dis. 2026 Sep 21;24:10.18332/tid/226881. doi: 10.18332/tid/226881 (PMC13594414; doi:10.18332/tid/226881)
Supplement: Supplementary file 1 [file TID-24-169-s001.pdf]

Tobacco Induced Diseases

Supplementary file

© 2026 Cheng Z. et al.

DOI:
10.18332/tid/226881

The content has been provided by the author(s) and has not been reviewed, verified, or endorsed by European Publishing. It may not have undergone peer review. The views, opinions, and recommendations expressed are solely those of the author(s) and do not necessarily reflect the position of European Publishing. European Publishing accepts no responsibility or liability for any consequences arising from the use of, or reliance on, this content.

Figure S1. Temporal trends in the number and age-standardized rate of smoking-attributable digestive disease deaths and DALYs by sex in China and the United States, 1990–2023

(A) Males, deaths. (B) Males, DALYs. (C) Females, deaths. (D) Females, DALYs. Bar charts represent the absolute number of cases (left y-axis) with error bars indicating 95% UIs, while line plots with shaded ribbons depict age-standardized rates per 100,000 population (right y-axis) with 95% UIs, for China and the United States from 1990 to 2023. Abbreviations: ASR, age-standardized rate; UI, uncertainty interval; DALYs, disability-adjusted life years.

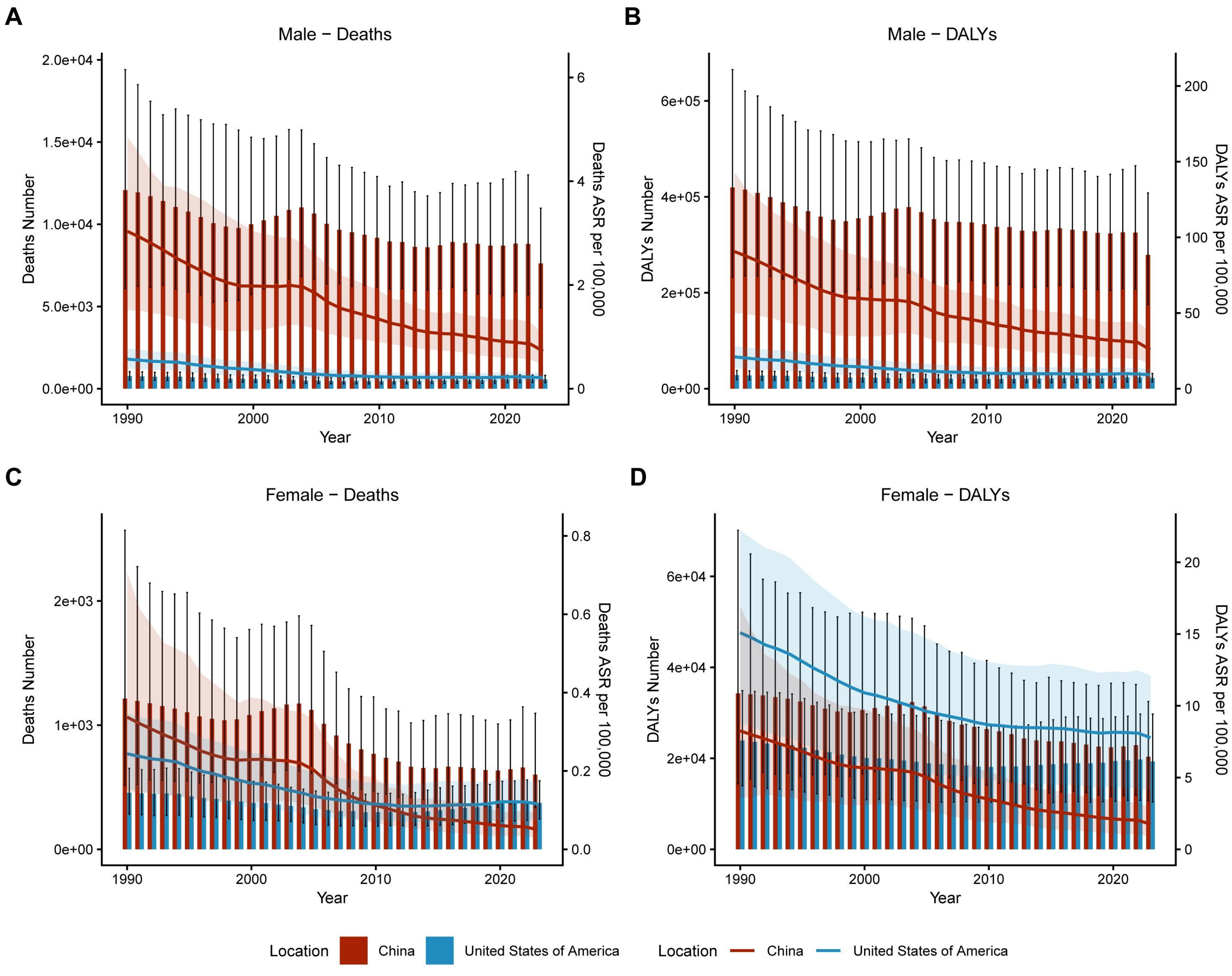

A
Male − Deaths
Deaths Number
Deaths ASR per 100,000
Year
B
Male − DALYs
DALYs Number
DALYs ASR per 100,000
Year
C
Female − Deaths
Deaths Number
Deaths ASR per 100,000
Year
D
Female − DALYs
DALYs Number
DALYs ASR per 100,000
Year
Location China United States of America
Location China United States of America

Figure S2. Joinpoint regression analysis of age-standardized rates of smoking-attributable digestive disease deaths and DALYs in China and the United States, 1990–2023

(A) Both sexes, deaths. (B) Both sexes, DALYs. (C) Males, deaths. (D) Males, DALYs. (E) Females, deaths. (F) Females, DALYs. Open circles represent observed age-standardized rates for China and solid triangles for the United States. Colored line segments (solid for China, dashed for the United States) indicate joinpoint regression model-fitted trends for each identified segment. The annual percentage change (APC) for each segment and the overall average annual percentage change (AAPC) for 1990–2023 are annotated, with asterisks (*) denoting statistical significance ($p < 0.05$). Joinpoint locations are labeled at the year of trend change. Abbreviations: ASR, age-standardized rate; APC, annual percentage change; AAPC, average annual percentage change; DALYs, disability-adjusted life years.

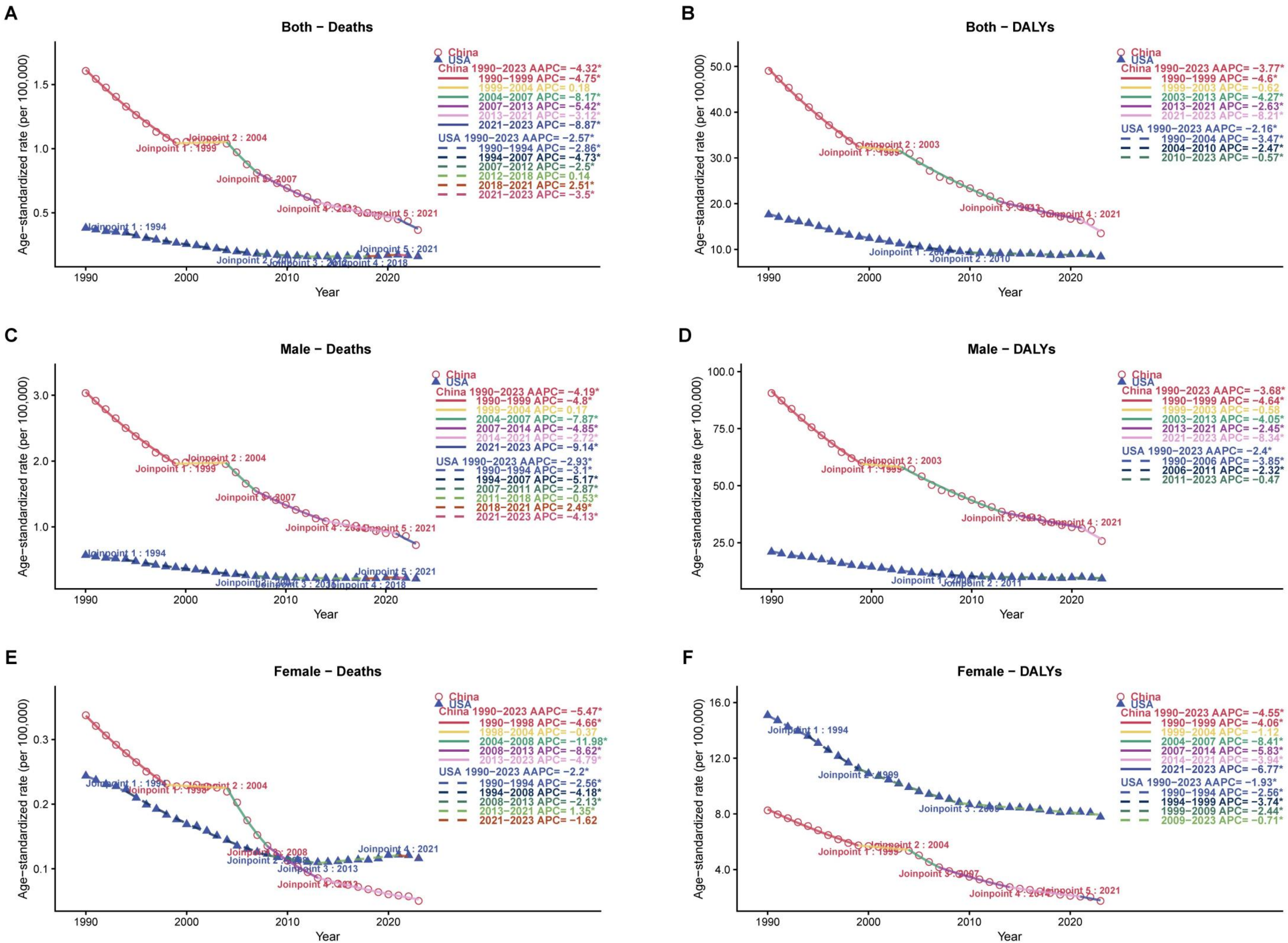
A
Both − Deaths
China
USA
China 1990−2023 AAPC= −4.32*
1990−1999 APC= −4.75*
1999−2004 APC= 0.18
2004−2007 APC= −8.17*
2007−2013 APC= −5.42*
2013−2021 APC= −3.12*
2021−2023 APC= −8.87*
USA 1990−2023 AAPC= −2.57*
1990−1994 APC= −2.86*
1994−2007 APC= −4.73*
2007−2012 APC= −2.5*
2012−2018 APC= 0.14
2018−2021 APC= 2.51*
2021−2023 APC= −3.5*
Joinpoint 1 : 1999
Joinpoint 2 : 2004
Joinpoint 5 : 2021
Joinpoint 1 : 1994
Joinpoint 5 : 2021
Joinpoint 4 : 2018
Age−standardized rate (per 100,000)
Year
B
Both − DALYs
China
USA
China 1990−2023 AAPC= −3.77*
1990−1999 APC= −4.6*
1999−2003 APC= 0.62
2003−2013 APC= −4.27*
2013−2021 APC= −2.63*
2021−2023 APC= −8.21*
USA 1990−2023 AAPC= −2.16*
1990−2004 APC= −3.47*
2004−2010 APC= −2.47*
2010−2023 APC= −0.57*
Joinpoint 2 : 2003
Joinpoint 4 : 2021
Joinpoint 2 : 2010
Age−standardized rate (per 100,000)
Year
C
Male − Deaths
China
USA
China 1990−2023 AAPC= −4.19*
1990−1999 APC= −4.8*
1999−2004 APC= 0.17
2004−2007 APC= −7.87*
2007−2014 APC= −4.85*
2014−2021 APC= −2.72*
2021−2023 APC= −9.14*
USA 1990−2023 AAPC= −2.93*
1990−1994 APC= −3.1*
1994−2007 APC= −5.17*
2007−2011 APC= −2.87*
2011−2018 APC= −0.53*
2018−2021 APC= 2.49*
2021−2023 APC= −4.13*
Joinpoint 1 : 1999
Joinpoint 2 : 2004
Joinpoint 5 : 2021
Joinpoint 5 : 2021
Joinpoint 4 : 2018
Age−standardized rate (per 100,000)
Year
D
Male − DALYs
China
USA
China 1990−2023 AAPC= −3.68*
1990−1999 APC= −4.64*
1999−2003 APC= −0.58
2003−2013 APC= −4.05*
2013−2021 APC= −2.45*
2021−2023 APC= −8.34*
USA 1990−2023 AAPC= −2.4*
1990−2006 APC= −3.85*
2006−2011 APC= −2.32*
2011−2023 APC= −0.47
Joinpoint 2 : 2003
Joinpoint 4 : 2021
Age−standardized rate (per 100,000)
Year
E
Female − Deaths
China
USA
China 1990−2023 AAPC= −5.47*
1990−1998 APC= −4.66*
1998−2004 APC= −0.37
2004−2008 APC= −11.98*
2008−2013 APC= −8.62*
2013−2023 APC= −4.79*
USA 1990−2023 AAPC= −2.2*
1990−1994 APC= −2.56*
1994−2008 APC= −4.18*
2008−2013 APC= −2.13*
2013−2021 APC= 1.35*
2021−2023 APC= −1.62
Joinpoint 2 : 2004
Joinpoint 3 : 2013
Joinpoint 4 : 2021
Age−standardized rate (per 100,000)
Year
F
Female − DALYs
China
USA
China 1990−2023 AAPC= −4.55*
1990−1999 APC= −4.06*
1999−2004 APC= −1.12
2004−2007 APC= −8.41*
2007−2014 APC= −5.83*
2014−2021 APC= −3.94*
2021−2023 APC= −6.77*
USA 1990−2023 AAPC= −1.93*
1990−1994 APC= −2.56*
1994−1999 APC= −3.74*
1999−2009 APC= −2.44*
2009−2023 APC= −0.71*
Joinpoint 1 : 1994
Joinpoint 2 : 2004
Joinpoint 5 : 2021
Age−standardized rate (per 100,000)
Year

Figure S3. Age-specific comparison of the number of smoking-attributable digestive disease deaths and DALYs between China and the United States in 2023

(A) Number of deaths by age group. (B) Number of DALYs by age group. Dumbbell plots display the values for China and the United States across fourteen age groups (30–34 to 95 plus years) in both sexes combined, with connecting lines indicating the magnitude of between-country differences. Abbreviations: DALYs, disability-adjusted life years.

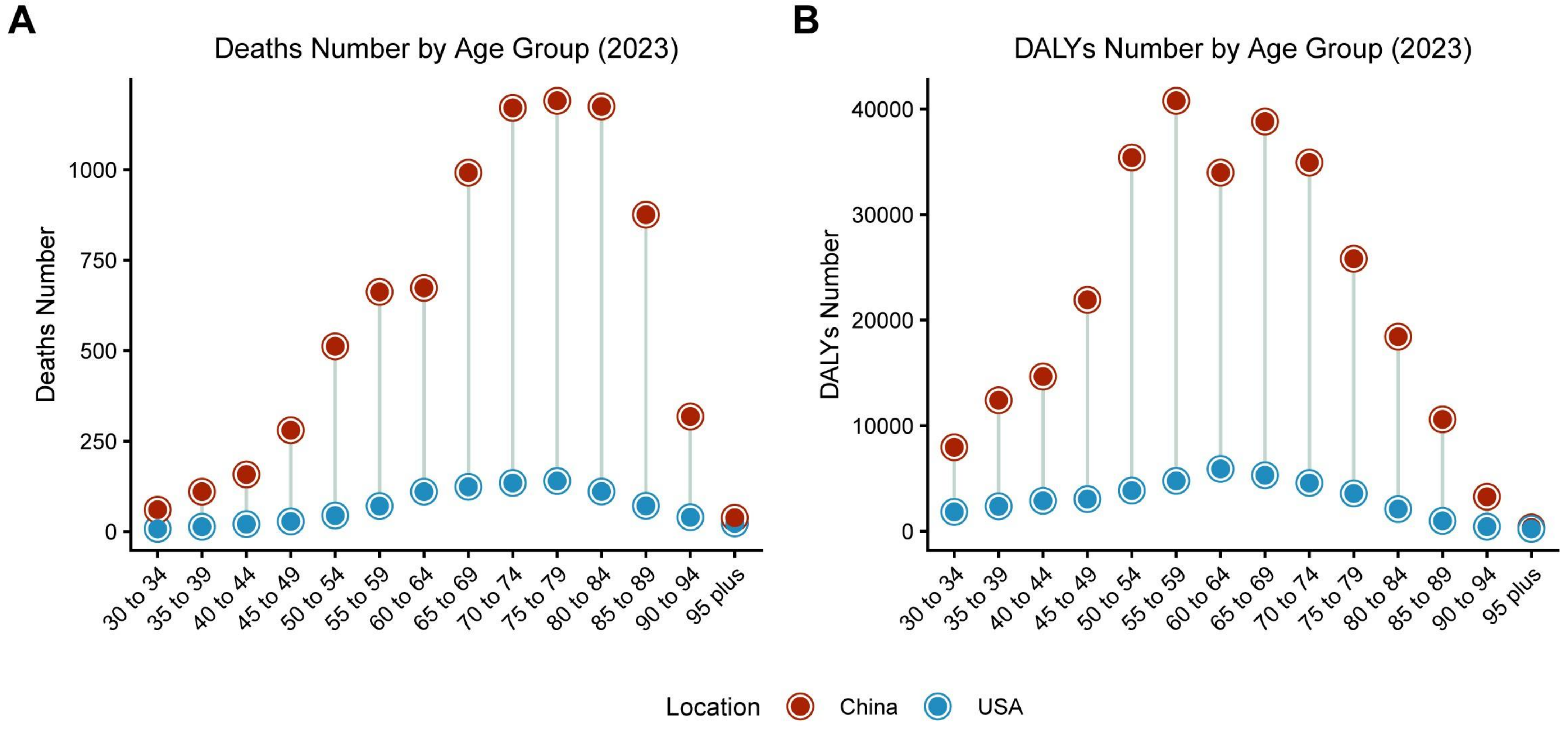

A
Deaths Number by Age Group (2023)
Deaths Number
0
250
500
750
1000
B
DALYs Number by Age Group (2023)
DALYs Number
0
10000
20000
30000
40000
30 to 34
35 to 39
40 to 44
45 to 49
50 to 54
55 to 59
60 to 64
65 to 69
70 to 74
75 to 79
80 to 84
85 to 89
90 to 94
95 plus
Location
China
USA

Figure S4. Bayesian age-period-cohort projections of smoking-attributable digestive disease burden from 1990 to 2035 in China and the United States

(A) Age-standardized death rate. (B) Age-standardized DALY rate. (C) Number of deaths. (D) Number of DALYs. Solid lines represent observed trends from 1990 to 2023, and projected estimates from 2024 to 2035 are shown with point markers and shaded ribbons indicating 95% prediction intervals. A vertical dashed line at 2023 separates the observed from projected periods. Results are stratified by country and sex (both sexes, male, and female). Abbreviations: ASR, age-standardized rate; DALYs, disability-adjusted life years; BAPC, Bayesian age-period-cohort.

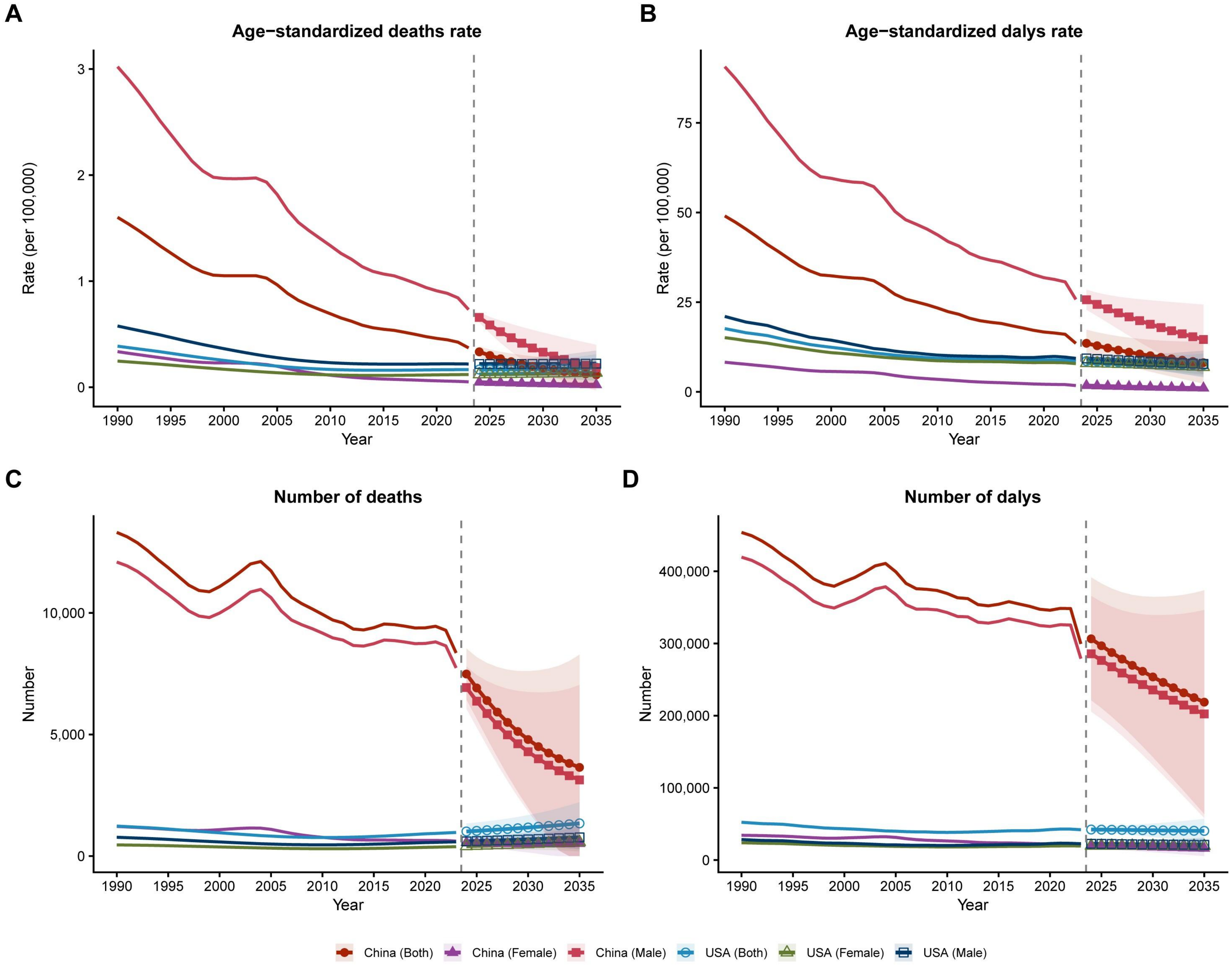
A
Age−standardized deaths rate
Rate (per 100,000)
Year
B
Age−standardized dalys rate
C
Number of deaths
Number
D
Number of dalys
China (Both)
China (Female)
China (Male)
USA (Both)
USA (Female)
USA (Male)

Table S1. Age-specific burden of smoking-attributable digestive diseases in China and the United States, 1990 and 2023

This table displays the age-specific number of cases (with 95% UI) and rates per 100,000 (with 95% UI) for deaths and DALYs attributable to smoking-related digestive diseases in both sexes combined, across fourteen age groups (30–34 to 95 plus years), with percentage changes from 1990 to 2023 and the estimated annual percentage change (EAPC) with 95% CI for each age group. Abbreviations: UI, uncertainty interval; EAPC, estimated annual percentage change; CI, confidence interval; DALYs, disability-adjusted life years.

Location	Measure	Age Group	Cases in 1990, n (95% UI)	Cases in 2023, n (95% UI)	Change in Cases (%)	Rate in 1990 (95% UI)	Rate in 2023 (95% UI)	Change in Rate (%)	EAPC (95% CI)
China	Deaths	30 to 34	292 (136 to 484)	61 (32 to 111)	-79.25	0.33 (0.15 to 0.55)	0.06 (0.03 to 0.1)	-82.91	-5.48 (-5.81 to -5.14)
China	Deaths	35 to 39	473 (232 to 750)	110 (54 to 194)	-76.66	0.52 (0.25 to 0.82)	0.09 (0.05 to 0.16)	-81.93	-5.01 (-5.25 to -4.77)
China	Deaths	40 to 44	648 (319 to 1070)	158 (79 to 270)	-75.64	0.96 (0.47 to 1.59)	0.17 (0.09 to 0.3)	-81.98	-4.98 (-5.19 to -4.76)
China	Deaths	45 to 49	810 (412 to 1360)	280 (149 to 470)	-65.44	1.57 (0.8 to 2.63)	0.28 (0.15 to 0.47)	-82.07	-4.94 (-5.09 to -4.78)
China	Deaths	50 to 54	1212 (596 to 2032)	512 (287 to 893)	-57.75	2.53 (1.25 to 4.25)	0.43 (0.24 to 0.75)	-82.93	-4.88 (-5.08 to -4.68)

China	Deaths	55 to 59	1544 (795 to 2544)	662 (375 to 1131)	-57.09	3.55 (1.83 to 5.85)	0.56 (0.32 to 0.96)	-84.21	-5.34 (-5.54 to -5.14)
China	Deaths	60 to 64	1749 (879 to 2725)	674 (389 to 1093)	-61.49	4.93 (2.48 to 7.69)	0.83 (0.48 to 1.34)	-83.23	-5.03 (-5.15 to -4.91)
China	Deaths	65 to 69	2142 (1107 to 3294)	992 (573 to 1609)	-53.68	7.81 (4.04 to 12.02)	1.3 (0.75 to 2.11)	-83.36	-5.05 (-5.25 to -4.85)
China	Deaths	70 to 74	1925 (1035 to 3169)	1171 (695 to 1873)	-39.18	10.2 (5.48 to 16.79)	1.92 (1.14 to 3.07)	-81.2	-4.78 (-5.03 to -4.54)
China	Deaths	75 to 79	1377 (725 to 2245)	1191 (675 to 1913)	-13.54	12.05 (6.34 to 19.64)	3.24 (1.84 to 5.2)	-73.15	-3.55 (-3.85 to -3.25)
China	Deaths	80 to 84	757 (382 to 1329)	1175 (627 to 1822)	55.11	13.56 (6.85 to 23.8)	5.23 (2.79 to 8.11)	-61.44	-2.58 (-2.83 to -2.33)
China	Deaths	85 to 89	312 (146 to 556)	876 (472 to 1480)	180.75	15.63 (7.29 to 27.87)	7.02 (3.78 to 11.86)	-55.07	-1.97 (-2.22 to -1.72)
China	Deaths	90 to 94	42 (20 to 75)	318 (168 to 535)	663.55	11.06 (5.43 to 19.95)	7.02 (3.7 to 11.81)	-36.51	-0.7 (-1.16 to -0.24)
China	Deaths	95 plus	12 (4 to 25)	38 (14 to 95)	232.09	17.66 (6.57 to 37.61)	3.09 (1.17 to 7.69)	-82.49	-5.86 (-6.51 to -5.21)
China	DALY s	30 to 34	21436 (11829 to 33152)	7952 (4414 to 13073)	-62.91	24.22 (13.37 to 37.46)	7.4 (4.11 to 12.17)	-69.44	-3.41 (-3.57 to -3.26)
China	DALY s	35 to 39	31822 (18279 to 47963)	12410 (7204 to 19642)	-61	34.75 (19.96 to 52.38)	10.5 (6.09 to 16.61)	-69.79	-3.3 (-3.42 to -3.18)
China	DALY s	40 to 44	38045 (20122 to 59280)	14658 (8453 to 22731)	-61.47	56.54 (29.9 to 88.1)	16.12 (9.29 to 24.99)	-71.49	-3.41 (-3.54 to -3.27)
China	DALY s	45 to 49	42035 (23788 to 67332)	21922 (13593 to 32796)	-47.85	81.22 (45.96 to 130.09)	21.98 (13.63 to 32.88)	-72.94	-3.57 (-3.71 to -3.43)

China	DALYs	50 to 54	55239 (31518 to 89340)	35407 (20955 to 53389)	-35.9	115.47 (65.88 to 186.75)	29.91 (17.7 to 45.09)	-74.1	-3.55 (-3.75 to -3.35)
China	DALYs	55 to 59	61806 (34230 to 98081)	40782 (24163 to 60528)	-34.02	142.09 (78.69 to 225.48)	34.5 (20.44 to 51.2)	-75.72	-3.88 (-4.04 to -3.73)
China	DALYs	60 to 64	59664 (32609 to 94147)	33976 (20607 to 52530)	-43.05	168.29 (91.98 to 265.56)	41.73 (25.31 to 64.51)	-75.21	-3.81 (-3.91 to -3.7)
China	DALYs	65 to 69	60236 (32768 to 93096)	38814 (23553 to 60287)	-35.56	219.75 (119.54 to 339.63)	50.86 (30.86 to 79)	-76.85	-3.98 (-4.11 to -3.84)
China	DALYs	70 to 74	43940 (24197 to 70027)	34936 (21506 to 54867)	-20.49	232.78 (128.18 to 370.97)	57.22 (35.22 to 89.86)	-75.42	-3.87 (-4.03 to -3.7)
China	DALYs	75 to 79	24870 (13258 to 38771)	25817 (14958 to 39858)	3.81	217.64 (116.02 to 339.3)	70.18 (40.66 to 108.35)	-67.76	-2.93 (-3.18 to -2.67)
China	DALYs	80 to 84	10708 (5465 to 18529)	18432 (10477 to 28379)	72.13	191.76 (97.88 to 331.82)	82.05 (46.64 to 126.33)	-57.21	-2.17 (-2.39 to -1.95)
China	DALYs	85 to 89	3556 (1727 to 6184)	10592 (5740 to 17657)	197.86	178.16 (86.51 to 309.81)	84.93 (46.02 to 141.57)	-52.33	-1.72 (-1.94 to -1.51)
China	DALYs	90 to 94	406 (199 to 714)	3263 (1750 to 5224)	703.07	107.89 (52.85 to 189.72)	72.05 (38.65 to 115.37)	-33.22	-0.47 (-0.91 to -0.03)
China	DALYs	95 plus	106 (40 to 221)	370 (139 to 890)	247.85	162.47 (60.91 to 337.95)	29.8 (11.17 to 71.71)	-81.66	-5.67 (-6.28 to -5.06)

USA	Deaths	30 to 34	15 (11 to 21)	8 (5 to 12)	-50.97	0.07 (0.05 to 0.09)	0.03 (0.02 to 0.05)	-53.6	-1.78 (-2.54 to -1.01)
USA	Deaths	35 to 39	25 (17 to 33)	14 (8 to 20)	-45.35	0.12 (0.08 to 0.16)	0.06 (0.04 to 0.09)	-50.66	-2.49 (-3.24 to -1.72)
USA	Deaths	40 to 44	35 (25 to 47)	21 (13 to 31)	-41.51	0.2 (0.14 to 0.26)	0.09 (0.06 to 0.14)	-51.77	-2.95 (-3.55 to -2.35)
USA	Deaths	45 to 49	46 (33 to 62)	28 (18 to 41)	-39.46	0.33 (0.23 to 0.44)	0.14 (0.09 to 0.21)	-57.31	-3.05 (-3.37 to -2.72)
USA	Deaths	50 to 54	61 (42 to 81)	44 (28 to 65)	-26.78	0.53 (0.37 to 0.7)	0.21 (0.14 to 0.32)	-59.55	-2.75 (-3 to -2.5)
USA	Deaths	55 to 59	85 (59 to 114)	71 (43 to 104)	-16.52	0.81 (0.56 to 1.09)	0.34 (0.21 to 0.5)	-57.59	-2.37 (-2.77 to -1.97)
USA	Deaths	60 to 64	127 (86 to 173)	110 (72 to 160)	-13.2	1.19 (0.81 to 1.62)	0.52 (0.34 to 0.75)	-56.6	-2.7 (-3.2 to -2.19)
USA	Deaths	65 to 69	174 (119 to 239)	124 (75 to 189)	-28.88	1.73 (1.18 to 2.37)	0.64 (0.39 to 0.98)	-62.7	-3.22 (-3.69 to -2.75)
USA	Deaths	70 to 74	209 (144 to 289)	134 (84 to 204)	-35.77	2.59 (1.78 to 3.57)	0.87 (0.54 to 1.31)	-66.52	-3.52 (-3.97 to -3.05)
USA	Deaths	75 to 79	212 (140 to 292)	140 (92 to 198)	-34.07	3.46 (2.28 to 4.76)	1.23 (0.81 to 1.75)	-64.36	-3.28 (-3.67 to -2.89)
USA	Deaths	80 to 84	155 (97 to 222)	111 (69 to 170)	-28.74	3.95 (2.46 to 5.64)	1.59 (0.99 to 2.45)	-59.58	-3.02 (-3.35 to -2.68)
USA	Deaths	85 to 89	51 (28 to 88)	71 (44 to 111)	39.34	2.51 (1.36 to 4.3)	1.87 (1.17 to 2.92)	-25.62	-1.9 (-2.26 to -1.54)
USA	Deaths	90 to 94	17 (9 to 33)	40 (22 to 66)	131.51	2.37 (1.21 to 4.51)	2.31 (1.28 to 3.83)	-2.6	0.05 (-0.04 to 0.14)
USA	Deaths	95 plus	10 (5 to 19)	23 (11 to 39)	126.35	4.48 (2.15 to 8.63)	3.6 (1.72 to 6.21)	-19.67	-0.87 (-1.19 to -0.55)

USA	DALYs	30 to 34	2975 (1735 to 4534)	1831 (861 to 3073)	-38.46	13.32 (7.77 to 20.3)	7.76 (3.65 to 13.02)	-41.77	-0.82 (-1.13 to -0.51)

USA	DALYs	35 to 39	3658 (2188 to 5483)	2360 (1213 to 3668)	-35.49	17.96 (10.74 to 26.91)	10.46 (5.37 to 16.25)	-41.76	-1.55 (-1.81 to -1.27)
USA	DALYs	40 to 44	4311 (2523 to 6354)	2880 (1408 to 4478)	-33.19	23.86 (13.97 to 35.17)	13.14 (6.43 to 20.43)	-44.92	-1.95 (-2.17 to -1.72)
USA	DALYs	45 to 49	4507 (2652 to 6460)	3045 (1507 to 4861)	-32.43	32.2 (18.95 to 46.15)	15.34 (7.59 to 24.49)	-52.35	-2.37 (-2.52 to -2.22)
USA	DALYs	50 to 54	4662 (2831 to 6470)	3849 (1949 to 6453)	-17.45	40.64 (24.68 to 56.4)	18.53 (9.38 to 31.07)	-54.4	-2.38 (-2.55 to -2.21)
USA	DALYs	55 to 59	5216 (3180 to 7391)	4771 (2456 to 7621)	-8.53	49.63 (30.26 to 70.32)	23.06 (11.87 to 36.84)	-53.53	-2.24 (-2.51 to -1.96)
USA	DALYs	60 to 64	6231 (3866 to 8986)	5901 (3486 to 8436)	-5.3	58.48 (36.28 to 84.33)	27.69 (16.36 to 39.58)	-52.65	-2.42 (-2.76 to -2.08)
USA	DALYs	65 to 69	6659 (4266 to 9344)	5303 (3005 to 8245)	-20.37	66.08 (42.33 to 92.72)	27.6 (15.64 to 42.91)	-58.23	-2.72 (-3.03 to -2.41)
USA	DALYs	70 to 74	6049 (3937 to 8558)	4568 (2687 to 7173)	-24.49	74.81 (48.68 to 105.83)	29.44 (17.32 to 46.23)	-60.65	-2.82 (-3.15 to -2.49)
USA	DALYs	75 to 79	4634 (2896 to 6526)	3576 (2248 to 5206)	-22.83	75.57 (47.23 to 106.41)	31.52 (19.81 to 45.89)	-58.29	-2.64 (-2.93 to -2.35)
USA	DALYs	80 to 84	2534 (1527 to 3668)	2097 (1235 to 3365)	-17.24	64.36 (38.77 to 93.15)	30.21 (17.8 to 48.48)	-53.06	-2.43 (-2.7 to -2.15)
USA	DALYs	85 to 89	622 (335 to 1041)	978 (580 to 1478)	57.19	30.57 (16.48 to 51.16)	25.65 (15.2 to 38.77)	-16.09	-1.47 (-1.81 to -1.12)
USA	DALYs	90 to 94	170 (87 to 322)	431 (240 to 694)	153.76	23.44 (11.98 to 44.51)	25.02 (13.96 to 40.33)	6.76	0.35 (0.25 to 0.46)
USA	DALYs	95 plus	91 (43 to 175)	220 (106 to 384)	141.94	40.75 (19.47 to 78.64)	34.99 (16.87 to 61.12)	-14.13	-0.66 (-0.98 to -0.35)

1
